# Specialist Physicians’ Attitude towards Emergency Medicine; a Semi-Structured Qualitative Study

**Published:** 2018-01-16

**Authors:** Shahrooz Tabrizi, Amir Nejati, Saharnaz Nedjat, Seyed Mojtaba Aghili

**Affiliations:** 1 Department of Emergency medicine, Imam Khomeini Hospital, Tehran University of Medical Sciences, Tehran, Iran.; 2 Department of Epidemiology, School of Public Health, Tehran University of Medical Sciences, Tehran, Iran.

**Keywords:** Emergency medicine, emergency service, hospital, attitude of health personnel

## Abstract

**Introduction:**

The present study is a survey to assess the pros and cons of emergency medicine (EM) from the viewpoint of the scholars from other medicine disciplines to improve the efficiency of EM in the healthcare system.

**Methods:**

This is a semi-structured qualitative study. Face-to-face interviews with various physicians with different specialties were performed to gather information on their viewpoints. Study population was selected mainly based on their history of collaboration with emergency medicine specialists in several educational hospitals in Tehran, Iran. All interviews were recorded and then transcribed to paper. Data were mainly categorized and reported into four themes: 1) general aspects of emergency medicine, goals and policies 2) Management of emergency department 3) Educational aspects 4) therapeutic aspects.

**Results:**

22 specialist physicians with the mean age of 47.3±7.6 years were studied (77.3% male). The average of their work experience as a specialist was 13.6±7.5 years. From the viewpoint of other experts, the establishment of EM and training of EM specialists is accompanied with relative disadvantages and advantages regarding goals and policies, patient management, therapeutic interventions and student education in the emergency department. Initiating resuscitation and maintaining hemodynamic stability and appropriate triage of the patients can add to the benefits of EM by preventing unreasonable hospitalization, and reducing the workload and difficulty of the work of other professionals working in the hospital.

**Conclusions:**

Based on the results of the current study, it seems that most Iranian specialist physicians have a positive attitude towards emergency medicine and think that emergency medicine could have beneficial effects for the health system and hospital management system.

## Introduction:

Launching a medical project in any region of the world is important due to its specific social, economic, and cultural requirements. In addition to its effects on the society, it specially affects other activists in the related field. Now that over a decade has passed since emergency medicine (EM) was launched in the Iranian health system; it is an opportunity to explore its various aspects (1-3). Considering the relatively new characteristics of EM field in the Iranian health care system, reviewing the opinion of other medical disciplines about EM, may be useful for governmental health policy makers and could be referred for reasonable distribution of resources (4, 5). Apparently, EM is past its deployment stages in Iran and has stepped into the stage of institutionalization, so it directly affects the society’s health, and its enforcement impact has been revealed (6-12).

Considering what was mentioned above, we decided to ask about the attitudes of other specialist physicians regarding this field of medicine. The present study is a survey to assess the pros and cons of EM from the view point of the scholars from other medicine disciplines to improve the efficiency of EM in the healthcare system.

## Methods:

***Study design and setting***

This is a semi-structured study had been conducted as a qualitative research from 2014 until 2016 in Tehran, Iran. The study protocol was approved by emergency medicine department research council and ethics committee of Tehran University of Medical Sciences. The researchers committed themselves to maintaining the principles of secrecy, and also did not any deliberate or unconscious censorship of the interviewees' statements. They were ensured that data will be recorded and typed confidentially without the participants’ name and solely on the basis of an encoded data. 

***Participants***

Convenience sampling was carried out. Study population included different faculties and specialists working in 3 educational hospitals affiliated to Tehran University of Medical Sciences. The specialists who had the most contact with the emergency department were included. Based on this decision, radiology, pathology, skin, radiotherapy and rehabilitation departments were excluded. Also due to the decision for evaluation of adults in the surgical and internal parts of emergency department, gynecologists and pediatricians were also excluded from the study. The names of the departments of each hospital were written on separate papers, and placed inside three separate bags each representing one of the hospitals. Then, three pieces of paper were drawn from each bag and samples were consecutively selected from the doctors practicing in the department the name of which was drawn. After that, by making an appointment and explaining the aim of the study, the eligible ones were asked to participate if they were interested. Not willing to participate in the study was considered as exclusion criterion. Sample selection was continued until data saturation was reached.

***Data gathering***

Face-to-face interviews about 30-40 minutes long were held by the same interviewer in order to gather information. Interviews were recorded. The research was done in line with the principles introduced in the declaration of Helsinki regarding ethics. The interview method was semi-structured and open .They were asked to state their points of view about emergency medicine on the topics related to the goals of the current study.

The interview was done purposefully and based on the following questions.

- What are the benefits of Emergency Medicine?
- What are the disadvantages of Emergency Medicine?
- What is the importance of emergency medicine in the hospital?
- What is your attitude and view on the specialty of Emergency Medicine?
- What role can an EM specialist have in the emergency department?

All interviews were recorded and then transcribed to paper. Data were mainly categorized and reported in four themes: 1) General aspects of emergency medicine, goals and policies; 2) Management of emergency department; 3) Educational aspects of emergency medicine establishment; 4) Therapeutic interventions conducted by emergency medicine physicians. Each category was also divided into different sub-sections that will be explained separately in the results section.

***Statistical analysis***

Data analysis was done using thematic content analysis based on Krippendorff approach (data collection, data reduction, deduction, and analysis). To verify the accuracy of the data, member checking methods were used. To confirm the coding and increase the validity of the analysis, the text was given to the other two peers, as anonymous, and an understanding was reached regarding the concepts and the division of the categories. Participants’ comments in the semi-structured interviews were collected and recorded and then typed, coded and analyzed based on the Krippendorff approach. 

## Results

***Baseline characteristics of*** ***interviewees***

Out of the 48 specialists who were asked to participate in the project, 22 (45.8%) were willing to participate and entered the study. These 22 specialist physicians had the mean age of 47.3±7.6 years and the average of their work experience as a specialist was 13.6±7.5 years (77.3% male). Table 1 shows the frequency of specialties that participated in the study.

***Interviewees’ opinions***

**General aspects of emergency medicine, goals and policies**

***Advantages***

Half of the respondents acknowledged the fact that it is undeniable that the field of emergency medicine is useful and necessary (54.5%). Some professors who were interviewed stated that the presence of emergency medicine provides reassurance for other disciplines and reduces their stresses (18%). In addition, some interviewees emphasized that the presence of emergency medicine has reduced mortality rate and health care costs.

***Disadvantages***

Some of the interviewees stated that emergency medicine physicians’ consecutive shifts can results in fatigue and increase the likelihood of medical errors (9%). One of the interviewees believed that there is no defined and targeted policy regarding the education and the duties of this specialty. Some believed that because of the heavy duty and stresses of this specialty it is not suitable for female doctors (9%).

**Management of emergency department**

***Overall management***

Professors interviewed believed that the role of this discipline as the custodian of emergency department (ED) is undeniable and exclusive responsibility of critically ill patients should be given to emergency medicine specialists (18%).

***Admission***

Another point emphasized by various interviewees was the necessity of taking into account the indications of admission despite the presence of different side pressures (18%). Some participants believed that the responsibility of triage should be given to a doctor, not a nurse, so that management of the patients who are admitted could be done better (9%). The load of patients without an indication for admission to ED causes exhaustion of the nursing and lab staff and can consequently affect the treatment of patients who really need the emergency care.

***Disposition***

3 of the respondents emphasized the need to stabilize an unstable patient before being transferred to other services (13.6%). Another respondent also emphasized this point in some other way and stated that an unstable and high risk patient should not be transferred to the ward just because the ED is crowded. The interviewees believed that emergency medicine service has a unique role in disposition of the patients that other services refuse to admit them (13.6%).

***Interaction with other services***

Almost half of those interviewed believed that greater coordination and interaction need to be established between various services and emergency service (45%). Some have suggested that joint meetings between the services, common morning reports, joint protocols or common rounds in the emergency department could result in more coordination. Five of the professors pointed to the time of the consultation with different services, they believed that the consultation should be done in a timely manner so that the admitting service could be involved in management of the patients (22%).

**Educational aspects of emergency medicine establishment**

***Advantages***

Interviewees believed that the continued presence of faculty members in the emergency department has a positive impact on treatment of patients (27%). Additionally, some professors believed that the emergency department environment is suitable for practical and scientific education of students (9%).

***Disadvantages***

Some professors who were interviewed believed that improving residency training in this field is required. Also, some believed that the high load of admitted patients not only affects the training of emergency medicine residents but also residents in other disciplines.

**Therapeutic interventions conducted by emergency physicians**

***Advantages***

A major part of the interviewees emphasized that specialized treatment in a minimum of time by EM professionals improves emergency care provided for the patients in the emergency department. Special emphasis was once again on the attendance of the faculty of emergency medicine and also it was noted that due to the comprehensive view of the EM specialists the treatment of patients was associated with a significant improvement.

***Disadvantages***

Four professors pointed to the prolonged duration of treatment for some patients in levels 3 and 4 of triage in emergency room that in some cases leads to doing unnecessary measurements (18%). Some of those interviewed also stressed that due to the high false-positive results in some lab tests these unnecessary diagnostic tests should be avoided as much as possible.

## Discussion

Based on the results of the current study, it is likely that most Iranian specialist physicians have a positive attitude towards emergency medicine and think that emergency medicine could have beneficial effects on the health care and hospital management systems.

**Table 1:** Frequency of specialties participating in the study (n=22)

**Specialty**	**N (%)**
Internist	6 (27.3)
General surgeon	5 (22.7)
Infectious disease specialist	4 (18.3)
Cardiologist	2 (9.2)
Gastroenterologist	1 (4.5)
Oncologist	1 (4.5)
Endocrinologist	1 (4.5)
Orthopedic surgeon	1 (4.5)
Neurologist	1 (4.5)

EDs are usually overcrowded wards with unpredictable referrals. Therefore, they need a different and special approach that leads to establishment of emergency medicine policies in hospitals (13-15).

The interviewees suggest that many specialists in small towns can be replaced by EM specialists due to the generalized curriculum and increased functional capabilities in this discipline. Some authors believe that EM and national health system interactions in some areas leading to development of policies for improving public health. Existing literature also point to various problems that must be addressed in order to take full advantage of the existing and rapidly developing relations between EM and public health. The clear policies by ministry of health regarding establishment of EM in educational and non-educational hospitals would be valuable and influential (13, 16, 17).

 After launch of EM, emergency department performance indices (EDPI) were assessed in previous studies. It was reported that establishment of EM was able to improve ED performance indices such as time to triage and patient disposition (18, 19).

Presence of EM may affect the education of other medical disciplines and distance them from complicated and critical patients. To resolve this problem, simulation-based curriculum would have considerable benefits (20-23).

One of the interviewees believed that the therapeutic scope of emergency medicine should be determined by other services to prevent potential challenges. Apparently, preparing and approving a national curriculum, with respect to all other disciplines would be the sole solution in this regard (24, 25).

The findings of this study showed that from the viewpoint of other experts working in the educational hospital, the establishment of EM and training of EM specialists is accompanied with relative disadvantages and advantages in goals and policies, patient management, therapeutic interventions and student education in the emergency department. Initiating resuscitation and maintaining the hemodynamic stability and appropriate triage of the patients can add to the benefits of EM regarding prevention of unreasonable hospitalization, and reduction of the workload and complexity of the work of other professionals working in the hospital.

***Limitation***

Lack of cooperation and lack of clarity in the comments of the interviewed experts and colleagues, due to some professional restrictions, caused some executive problems, which we tried to reduce as much as possible by expressing the desired goals of the research. 

## Conclusion

Taken together, considering the points cited by interviewees it can be concluded that the scholars from other disciplines are well aware of the importance of the field of emergency medicine and there are many positive comments about this discipline and they believe that presence of this field has resulted in better management of ED. However, there are some criticisms of this discipline and the better coordination of EM with other services is called for.
